# Stress and 5-HTT (SLC6A4) as an indicator of suicidal behavior: a population study among the Dubla tribe of Daman

**DOI:** 10.1186/1755-8166-7-S1-P44

**Published:** 2014-01-21

**Authors:** Sweta Saha, Masan Kambo Newmei, Shivani Pasi, Piyoosh Kumar Singh, Vadlamudi Rao

**Affiliations:** 1 Department of Anthropology, University of Delhi, Delhi, India

**Keywords:** Stress, Suicide, 5HTT-VNTR, Tribe

## Background

Suicide is considered to be characteristic of modern societies and thought to be an alien feature in tribal societies. Few earlier studies have reported cases of suicide in tribal communities but there is a dearth of evidences from India and around the globe investigating suicide among the tribal communities. The stress diathesis model is the best framework to understand the complex mechanisms interacting throughout the relationship between stress and suicide. The present study aims to understand the genetically determined vulnerability of suicide under stressful life events among the Dubla tribe of Daman.

## Method

84 unrelated individuals were recruited for the study. Face to face interview was conducted to collect information. Columbia Suicide severity Rating Scale (C-SSRS) and Presumptive Stressful Life Event Scale (PSLES) was administered to measure suicidal behavior and stressful life events respectively. Alcohol abuse was assessed using DSM-IV based questionnaire. Sociodemographic variables were also recorded. Buccal cell samples were collected for genetic analysis. Genotyping of 5HTT-VNTR (Stin2) was done with 55 samples.

## Result

Bivariant analyses showed significant difference in stressful life events among suicide ideators and non-ideators (OR=8.625, p= 0.00007). Also, stressful life events were higher in suicide attempters both in males and females [OR=8.438, p= 0.02 (males); OR=9.454, p= 0.04 (females)]. The frequency of the 5HTT VNTR 10 repeat allele and12repeat allele was 27% and 73% respectively in the studied population. Significant difference was observed in 10 repeat allele (Stin 2.10) for suicide attempters and non attempters among females (odds ratio, OR = 4.263; p = 0.000003).

## Conclusion

Stress was found to be a major predictor of suicide among the Dubla tribe. 5HTT VNTR 10 repeat allele was associated with suicide attempt among females. This finding of 5-HTT genes associated with female suicide is justified by the dimorphic nature of the serotonergic system.

